# Immunological strategies against spike protein: Neutralizing antibodies and vaccine development for COVID‐19

**DOI:** 10.1002/ctm2.184

**Published:** 2020-10-14

**Authors:** Jiansheng Huang, Hui Huang, Dongdong Wang, Chuan Wang, Youfa Wang

**Affiliations:** ^1^ Department of Medicine Vanderbilt University Medical Center Nashville Tennessee; ^2^ Department of Biochemistry and Center of Structural Biology Vanderbilt University Nashville Tennessee; ^3^ Center for Metabolism, Obesity and Diabetes Research McMaster University Hamilton ON Canada; ^4^ Global Health Institute, School of Public Health Xi'an Jiaotong University Health Science Center Xi'an China

**Abbreviations**
ACE2: angiotensin‐converting enzyme 2
ADE: antibody‐dependent enhancement
BTK: Bruton tyrosine kinase
COVID‐19: novel coronavirus disease‐2019
RBD: receptor‐binding domain
S protein: spike glycoprotein
SARS: severe acute respiratory syndrome
SARS‐CoV‐2: severe acute respiratory syndrome coronavirus 2
SpO2peripheral capillary oxygen: peripheral capillary oxygen saturation

Dear Editor,

Coronavirus disease 2019 (COVID‐19), which is caused by infection of the severe acute respiratory syndrome coronavirus‐2 (SARS‐CoV‐2), is currently a devastating threat to public health. Accumulating evidence supports that severe COVID‐19 patients (peripheral capillary oxygen saturation (SpO2) ≤ 94%) may develop acute respiratory distress syndrome. Understanding the dysregulated host immune response to SARS‐CoV‐2 may enable us develop therapeutics for COVID‐19. Recent transcriptional analysis revealed that there was an imbalanced response with minimal levels of interferons and a remarkable increase of chemotactic and inflammatory response characterized by expression of IL‐1β, IL‐6, TNF, CXCL1 as shown in Figure 1.^1^ The excessive formation of proinflammatory cytokines, such as IL‐6 and IL‐1β, may result in a higher risk of vascular hyperpermeability and respiratory failure.^1^, ^2^ Recent research progress on mechanistic studies of SARS‐CoV‐2 and clinical trials of neutralizing antibodies and vaccine development is one giant leap. Here, we summarize the advantages and disadvantages of different strategies to develop effective vaccines. We also explore the viability and challenges in developing neutralizing antibodies for COVID‐19.

**FIGURE 1:**
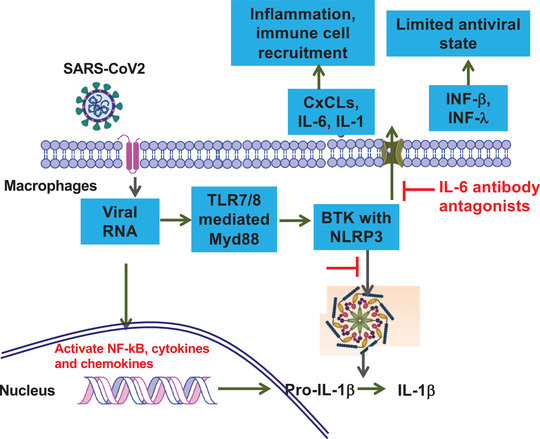
Potential mechanisms of SARS‐CoV‐2 to activate the NLRP3 inflammasome complex. SARS‐CoV‐2 may boost the production of cytokine release syndrome (CRS)‐related cytokines, such as IL‐1β and IL‐6. Viral RNA of the coronavirus activates TLR7/8‐Myd88 signaling and results in the upregulation of NLRP3 and interleukin‐1 beta (IL‐1β) through the activation of the transcription factor NF‐kB. It is revealed that there is dramatically increased Bruton tyrosine kinase (BTK) activity, as supported by autophosphorylation, and elevated levels of IL‐6 in blood monocytes from patients with severe COVID‐19 (peripheral capillary oxygen saturation (SpO2) ≤ 94%, clinicaltrials.gov/ct2/show/NCT04292899) compared to the healthy control.^1^ This implicates that targeting exaggerated host inflammation with a BTK inhibitor Acalabrutinib might be a therapeutic approach for patients with severe symptoms. A BTK inhibitor acalabrutinib can block the interaction of BTK and NLRP3 and exhibits early promise for treatment of COVID‐19

Antibodies that block the interaction between S protein and angiotensin‐converting enzyme 2 (ACE2) may effectively prevent the infection of SARS‐CoV‐2 as shown in Figure 2A. The neutralizing antibody CR3022 can bind with the S protein of SARS‐CoV‐2 as shown in Figure 2B.^3^ The monoclonal‐ neutralizing antibody CR3022 recognizes a highly conservative domain, which is distal from the receptor‐binding domain (RBD) of SARS‐CoV‐2 S protein.^4^ However, it is intriguing to confirm it by performing in vitro competitive binding assays to demonstrate how efficient CR3022 is competing with the binding of SARS‐CoV‐2 S protein to ACE2 and preventing the infection of cells with SARS‐CoV‐2 challenge. Another human monoclonal antibody S309 identified from a convalescent SARS patient was found to be able to bind with the S protein of SARS‐CoV‐2.^5^ The structure of S protein in complex with S309 is shown in Figure 2C and 2D. These studies allow the development of potent‐neutralizing antibodies through analysis of conformational change and chemical space restraints of the identified neutralizing antibody using molecular modeling pipelines.^6^ This opens the new field of using neutralizing antibody cocktails as a therapeutic approach for COVID‐19. The cocktail treatment with different antibodies will enhance the capacity to prevent the infection of SARS‐CoV‐2 and may reduce the occurrence of neutralization‐escape mutants.

**FIGURE 2:**
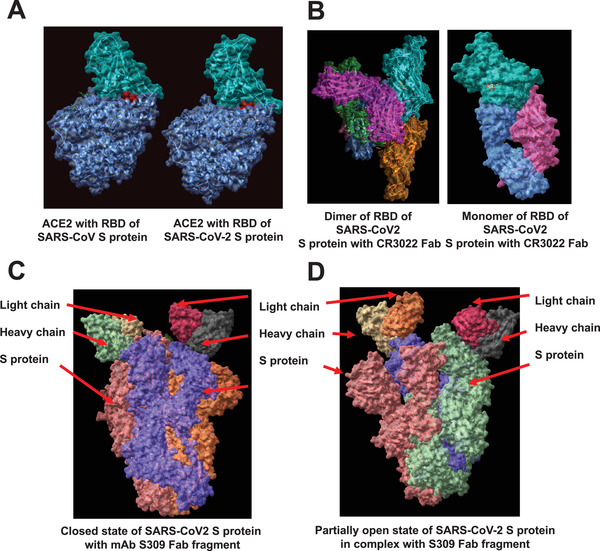
Complex of ACE2 with either RBD of SARS‐CoV S protein or RBD of SARS‐CoV‐2 S protein, complex of S protein of SARS‐CoV‐2 with neutralizing antibody CR3022 or S309. (A) The structure of RBD of SARS‐CoV S protein with ACE2 (PDB: 2AJF). ACE2 is shown as blue, RBD of the SARS‐CoV spike protein is highlighted in cyan. The α1 helix of the ACE2 mainly interacts with the RBD. Residue of Tyr442 of the RBD interacts with Asp 30 and His34 of ACE2, which are highlighted in red and the N‐linked glycosylation site is shown in green. The structure of S protein SARS‐CoV‐2 with ACE2 (PDB: 6M0J). ACE2 is shown as blue, surface representation “RBD” of the SARS‐CoV‐2 spike protein is highlighted in cyan, and the glycosylation site is shown in green. The structures demonstrate the interaction between ACE2 shown in blue. Residues of Lys417 and Leu 455 of the RBD interact with Asp30 and His34 of ACE2, which are highlighted in red. (B) Dimer of RBD of SARS‐CoV‐2 S protein with neutralizing antibody CR3022 (PDB: 6YLA) and monomer of RBD of SARS‐CoV‐2 S protein with CR3022 Fab (PDB: 6W41) are shown. RBD of S protein is highlighted in cyan or orange in dimer, the heavy chain of human antibody CR3002 Fab is shown as blue or green in dimer, and the light chain of CR3022 Fab is shown in pink or magenta in dimer. Structural data of RBD of SARS‐CoV‐2 S protein with CR3022 Fab was retrieved from Protein Databank. (C) Structure of SARS‐CoV‐2 S protein with neutralizing antibody S309 Fab fragment (PDB: 6WPS) is shown in close state. The S protein is highlighted in blue, pink, orange, the heavy chain of human antibody S309 is shown as dim grey or light sea green, and the light chain of S309 is shown in purple, brown. D. The structure of SARS‐CoV‐2 S protein with a neutralizing antibody S309 Fab fragment (PDB: 6WPT) is shown in close state open state. The S protein is highlighted in blue, pink, or light sea green, the heavy chain of human antibody S309 is shown as dim grey or brown, and the light chain of S309 is shown in purple or orange. Structural data of SARS‐CoV‐2 S protein with S309 Fab was retrieved from the Protein Databank. The interactive visualization of structures of the S protein of SARS‐CoV‐2 and antibody was performed using an extensible molecular modeling software UCSF Chimera

DNA vaccine, RNA vaccine, and vaccine with adenovirus encoding S proteins and inactivated SARS‐CoV‐2 vaccine are reported promisingly as shown in Table 1. INO‐4800, a synthetic DNA encoding the S protein, led to robust production of the S protein and induced the production of immune IgG. This effectively abolished the binding of the S protein to the ACE2 of host cells.^7^ Similarly, another DNA vaccine candidate expressing various types of S protein was evaluated in rhesus macaques.^8^ Interestingly, this DNA vaccine effectively enhanced humoral and cellular immune responses and reduced the median viral loads in the respiratory system.^8^ In addition, Moderna developed the first RNA vaccine candidate mRNA‐1273 reconstituted with lipid nanoparticles. mRNA‐1273 exhibited full protection against viral infection in the lung of a murine model.^9^ The safety of mRNA‐1273 was examined in the phase I clinical study. The adverse effects were temporary and self‐resolving.^9^ These studies prove the safety profile of the nucleic acid vaccines, and the efficacy needs to be examined in the phase II/III clinical trial.

**TABLE 1:** Potential vaccine candidates for COVID‐19 in clinical trials

Number	Vaccine candidates	Existing, licensed vaccine from the same platform	Antigens or epitope residues	Key strengths and limitations	Current stage of clinical trials	References
1.	DNA vaccines	No licensed vaccine	INO‐4800, DNA plasmid (S protein) vaccine from Inovio Pharmaceuticals.	No live contagious virus to be handled, easy scale‐up, low production costs, high heat stability, tested in humans for SARS‐CoV, rapid production possible. Vaccine needs specific delivery devices to reach good immunogenicity	Phase I NCT04336410 Phase I/II NCT04447781	^7^, ^8^
2.	RNA vaccines	No licensed vaccine	mRNA‐1273, Spike protein, LNP encapsulated mRNA from Moderna/NIAID	No contagious virus to be handled, vaccines are typically immunogenic, rapid production. Safety issues with reactogenicity	Phase III NCT04470427	^9^
3.	Nonreplicating viral vector	Adenovirus type 5 vector with the full‐length S protein, not only HIV and SIV but also multiple DNA and single‐ and double‐stranded RNA viruses	Ad5‐nCoV, CanSino Biological Inc./Beijing Institute of Biotechnology and the adenovirus‐vectored vaccine ChAdOx1 nCoV‐19 (AstraZeneca)	Targets mucosal inductive sites, infects dividing, nondividing, and dendritic cells; no integration, physically and genetically stable. Specific for nonreplicating vector: Safe, long history of gene therapy use multiple serotypes and chimeric forms, but high doses needed to elicit immunity	Phase II ChiCTR2000031781 Phase III Covid‐19 vaccine trial in Saudi Arabia (Ad5‐nCoV) and Phase III NCT04516746 (ChAdOx1)	^11^, ^12^
4.	Live attenuated vaccines	Yes, based on the established flu‐based DelNS1 live attenuated influenza virus (LAIV) platform. Express a specific antigen with flu vector to induce immunity targeting the RBD of S protein	Whole virus from Beijing Institute of Biological Products/Wuhan Institute of Biological Products or Sinovac	Activate an immune response that can protect against infection before the virus is killed by the immune system. Because the vaccine virus is replication‐competent, there is a risk of reversion to virulence after administration, albeit small. This approach may avoid ADE as observed in the experimental vaccine for SARS‐CoV	Preclinical	https://doi.org/10.1038/s41563-020-0746-0. net/news_cepi/cepi‐partners‐with‐university‐of‐hong‐kongto‐develop‐covid‐19‐vaccine/
5.	SARS‐CoV‐2‐rS	Spike protein subunit vaccine	Recombinant S protein, Navovox, Inc. Spike protein nanoparticle vaccine with Matrix‐M adjuvant	No contagious virus to be handled, adjuvants used to enhance immunogenicity	Phase II/III NCT04368988	ClinicalTrials.gov: NCT04368988
6.	Inactivated vaccines	Yes, inactivated poliovirus vaccine, whole cell pertussis (whooping cough) vaccine, rabies vaccine and the hepatitis A virus	PiCo vaccine, whole virus from Beijing Institute of Biological Products BBIBP‐CorV (Sinopharm/CNBG, China)	Very safe, without the risk of the virus or bacteria mutating back into its disease‐causing pathogen, can be freeze dried and easily stored	Phase I ChiCTR2000031809 NCT04352608 The phase III trial (PiCo) is underway in Brazil, The phase III trial of BBIBP‐CorV is underway in Abu Dhabi, UAE	^13^, ^14^

NVX‐CoV2373 was synthesized by a combination of the genetic sequence of SARS‐CoV‐2 with a proprietary Matrix‐M adjuvant. The vaccines induced immune responses and elicited high levels of neutralizing antibodies in a preclinical study.^10^ The phase I clinical trial demonstrated its safety profile. These studies demonstrate the potential of using the RBD domain in the SARS‐CoV‐2 for vaccine development and provide the preclinical evidence for the future clinical trials.^10^ The advantage of this strategy is that there is no contagious virus to be handled, and adjuvants can enhance immunogenicity. This platform has already been used to successfully develop effective vaccines for influenza and human papillomavirus (HPV). The weakness is that the large‐scale manufacturing capacity is limited, and the yield may not be sufficient for worldwide application.

The phase I clinical trial of an Ad5‐vectored SARS‐CoV‐2 vaccine was recently completed in Wuhan, China.^11^ No serious adverse event was observed, and there was significantly increased levels of neutralizing antibodies after vaccination.^11^ This important study demonstrates that the Ad5 vaccine induced the humoral response against SARS‐CoV‐2, and its efficacy needs to be examined in the future clinical study. In addition, another adenovirus‐vectored vaccine ChAdOx1 nCoV‐19 was developed to express the antigen S protein of SARS‐CoV‐2. This vaccine induced strong humoral and T cell‐involved immune response in rhesus macaques.^12^ More importantly, no pneumonia symptom was observed.^12^ The advantage of Ad5‐vectored vaccine is its robust ability to activate the immune response before the virus is inactivated by the human immune system; this promotes the efficacy of vaccines to protect against infection. In addition, the production platform has solid resources of gene therapy with multiple serotypes, but the down side is that high doses will be needed to induce the protective immune response.

In addition to the Ad5‐vectored COVID‐19 vaccine, an inactivated S protein of SARS‐CoV‐2 vaccine (PiCoVacc) was found to induce specific neutralizing antibodies.^13^ This vaccine was able to partially protect against the SARS‐CoV‐2 challenge in macaques.^13^ More importantly, there was no observable antibody‐dependent enhancement (ADE) effect after immunization. Another inactivated SARS‐CoV‐2 vaccine candidate (BBIBP‐CorV) was developed. A HB02 strain isolated from the COVID‐19 bronchoalveolar lavage samples was passaged to obtain an adapted strain with efficient replication capacity for high productivity before inactivation of the viral strain with β‐propionolactone. This strategy enables the elimination of viral infectivity and validates its stability. Importantly, this vaccine efficiently induced protective neutralizing antibodies in rhesus macaques.^14^ These results support the future assessment of its efficacy in human patients. These vaccine strategies have different strengths and limitations, but each offer some promise. Inactivated vaccine is very safe, can be freeze dried, and easily stored, and there is no risk of the virus mutating back into its disease‐causing pathogen. Both viral vector vaccines and recombinant S protein nanoparticle vaccines have higher safety profiles and are more immunogenic, but nonreplicating viral vector vaccines may have lower efficacy because of the existing immune response to the vector.

It is very challenging to develop an effective vaccine against COVID‐19 because of the property of single‐strand RNA. Although some of these vaccine candidates (inactivated vaccines and recombinant S protein vaccines) are produced on platforms that have been used for other virus vaccines, the efficacy and the duration of vaccine‐induced immune responses need to be determined. Given the high failure percentages and cost of vaccine development, a systemic platform with a linear sequence of experimental studies, data analysis, and manufacturing process is recommended for vaccine candidates. It is required to have a new pandemic paradigm to develop an effective vaccine rapidly. The worldwide vaccine production, procurement, and distribution should be considered before completing the clinical trials.

## CONFLICTS OF INTEREST

The authors declare no conflict of interest.

## AUTHOR CONTRIBUTIONS

J.H and Y.W wrote this review. H.H created the figures, and C.W and D.W edited the review.
